# The Relationship Between Cannabis Use and Schizophrenia As a Risk Factor or For Its Therapeutic Potential: A Systematic Review of Evidence

**DOI:** 10.7759/cureus.92793

**Published:** 2025-09-20

**Authors:** Jaisingh Rajput, Sandhya Narahari, Taha Arif, Rabiya Iftikhar, Turimula Arpan, Abdullah Tariq, Hamad Mohammad Ali Duleh, Sri Pranita Cherukuri

**Affiliations:** 1 Family Medicine, Baptist Health, Montgomery, USA; 2 Department of Medicine, Siddhartha Medical College, Vijayawada, IND; 3 Psychiatry, National Forensic Mental Health Services, Dublin, IRL; 4 Medicine, Our Lady's Hospital Navan, Navan, IRL; 5 Medicine, SVS Medical College, Mahbubnagar, IND; 6 Medicine, Amin Welfare and Teaching Hospital, Sialkot, PAK; 7 Medicine, Zhejiang University School of Medicine, Hangzhou, CHN; 8 Public Health, Columbia University Mailman School of Public Health, New York, USA

**Keywords:** cannabis, psychosis, schizophrenia, therapeutic potential, δ9-tetrahydrocannabinol (thc)

## Abstract

The rising use of cannabis in the medical field has prompted the revival of the global debate about its psychotropic effects, especially when it comes to schizophrenia, a debilitating mental disorder that afflicts approximately 20 million people around the globe. Although Δ9-tetrahydrocannabinol (THC) is commonly considered a risk factor for psychosis, cannabidiol (CBD) has been studied for its potential therapeutic use. This review aims to clarify the central controversy surrounding the question of whether cannabis worsens schizophrenia symptoms by presenting a selective analysis of the available scientific literature. A systematic review was performed according to PRISMA 2020 guidelines. PubMed, PubMed Central, Cochrane Library, and Google Scholar were searched for peer-reviewed studies published between 2017 and 2025. A total of 112 records were obtained in the first phase of the research process, and after an intense screening process, eight high-quality studies meeting the inclusion criteria were included based on rigorous quality evaluation (A MeaSurement Tool to Assess systematic Review 2 or AMSTAR 2, Cochrane Risk of Bias Tool, Newcastle-Ottawa Scale, and scale for the quality assessment of narrative review articles (SANRA)). Data were extracted and systematically synthesized across three analytical domains: THC as the cause of schizophrenia, the influence of THC on the course of symptoms, and the potential therapeutic effects of CBD. The results provided strong evidence that the risk of developing schizophrenia was significantly higher with the use of high-THC-content cannabis, particularly when used by patients with family vulnerability and by those who used cannabis regularly during adolescence. THC was also demonstrated to exacerbate not only positive and negative psychotic symptoms but also cognitive disturbances in subjects with established schizophrenia. Conversely, there was evidence for the antipsychotic and neuroprotective properties of CBD and its therapeutic potential. However, clinical evidence for CBD was still limited; most studies were small, and few had long-term follow-up. This review emphasizes the importance of distinguishing the components of cannabis in psychiatric discussions. Although THC has potent schizophrenia liability, CBD is still a novel but promising candidate for treatment. Large controlled clinical trials need to be a priority for future research to determine the therapeutic and mechanistic limits of cannabis in mental health. These implications are very important for the clinical arena, public health policy, and research agenda on the topic of cannabis use and schizophrenia treatment.

## Introduction and background

Mental health is an essential part of human health and well-being; it influences how people think, feel, behave, and deal with other people. Mental health problems have increased owing to greater social stressors, particularly economic insecurity, digital isolation, and psychosocial disintegration [1,2]. Coping with stress is one of the most frequently reported reasons for cannabis use, particularly as its use becomes more normalized in both recreational and medical contexts [3]. Dubbed as marijuana, weed, or pot, cannabis is currently one of the most frequently used psychoactive drugs in the world [4].

Over the recent years, the field of cannabis' legal status has been experiencing some crucial changes on a global basis; the most sudden changes happened in North America and to a lesser degree in some European states as well. Social perception has shifted to considering the drug harmless, which is why its use has now become normal in all age groups. There are over 100 cannabinoids in cannabis; Δ9-tetrahydrocannabinol (THC), among them, is the compound responsible for producing the ‘high,’ along with altered perceptions and impaired cognitive functioning. Cannabidiol (CBD), in turn, is not intoxicating and is becoming widely acknowledged to be anti-inflammatory and even antipsychotic [5,6].

Nonetheless, the overlap between cannabis use and schizophrenia has prompted numerous clinical and investigative apprehensions. Schizophrenia is a chronic mental illness that affects an estimated 20 million individuals worldwide. It is associated with positive symptoms (e.g., hallucinations and delusions), negative symptoms (e.g., affective flattening and social withdrawal), and cognitive deficits. The disorder commonly occurs in late adolescence or early adulthood, an age when cannabis consumption is also on the rise [7]. Epidemiological studies have demonstrated that the prevalence of cannabis use among adolescents has increased worldwide, from 7% in 2007 to 8.4% in 2017, with the U.S. populations showing the highest increase [8].

The Diagnostic and Statistical Manual of Mental Disorders (DSM)-5 classifies schizophrenia as a spectrum of psychotic disorders [9]. It is important to note that psychosis, a symptom of schizophrenia, can develop independently and without drug use. Cannabis and high-potency THC have emerged as the focus of discourses on psychiatric etiology and comorbidity [10]. Some researchers argue that cannabis can elicit and exacerbate schizophrenia, although others propose that even treatment with CBD could be helpful, especially for symptoms such as psychosis. These conflicting perspectives have led to the polarization of opinions in the scientific world [11,12].

**Figure 1:**
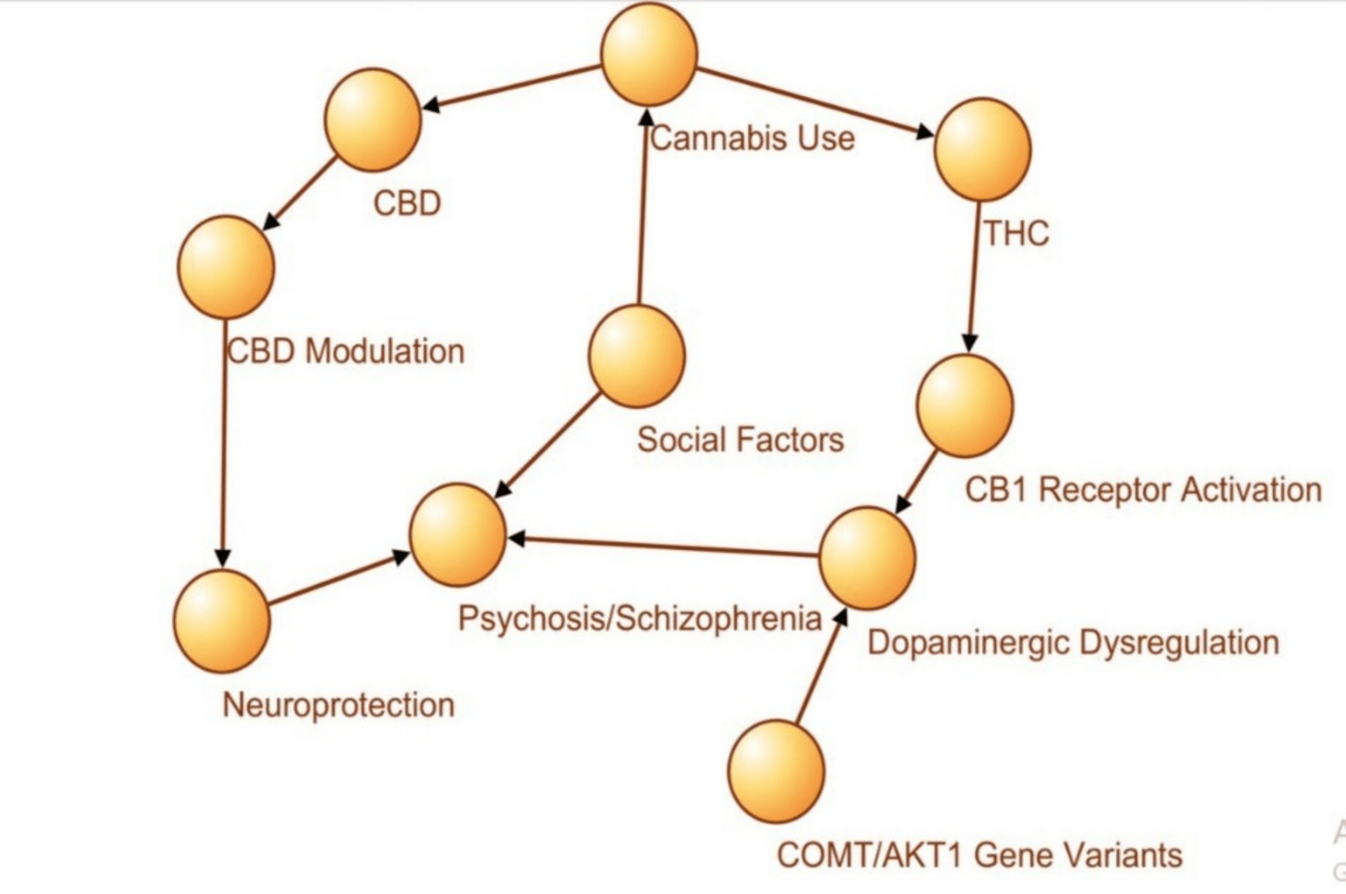
Theoretical framework: Biological, genetic, and social pathways linking cannabis to schizophrenia CBD: Cannabidiol; THC: Tetrahydrocannabinol; CB1: Cannabinoid receptor type 1; COMT/AKT1: Catechol-O-methyltransferase/Protein kinase B1 Image created by the authors on Microsoft Powerpoint (Microsoft Corp., Redmond, WA, US) [9-14]

To work beyond this binary paradigm, it is necessary to use a theoretical model that combines, in an organic way, the biological and genetic pathways that positively mediate as well as the social mechanisms by which cannabis contributes to the risk of developing schizophrenia. Several interacting factors determine the divergent effects of cannabis use (Figure 1).

Exposure to THC leads to a series of neurobiological perturbations, notably activation of cannabinoid receptor type 1 (CB1) receptors in the endocannabinoid system (ECS), which interferes with dopamine action and the pathophysiology of psychosis. This course carries significant risks, especially for those with known genetic vulnerabilities, such as variants in the catechol-o-methyltransferase (COMT) and AKT serine/threonine kinase 1 (AKT1) genes that influence dopamine breakdown and susceptibility to stress [13].

In contrast, CBD influences other neurochemical systems such as serotonin 5-Hydroxytryptamine (serotonin) receptor 1A (5-HT1A) receptors and glutamate homeostasis, with possible neuroprotective and antipsychotic properties. This risk landscape is further modulated by social risk factors, including early exposure to cannabis, peer influence, socioeconomic adversity, and trauma, which either augment the THC risk or diminish CBD protection [14]. Figure 1 shows the theoretical framework, the biological, genetic, and social pathways linking cannabis to schizophrenia.

The network model in Figure 1 depicts the integration of cannabis consumption (THC and CBD), neurobiological mechanisms (e.g., CB1 receptor activity and dopaminergic dysregulation), genetics (e.g., COMT and AKT1 polymorphisms), and social factors (e.g., early exposure and trauma). This model explains why the same cannabinoids interact differentially with negative and cognitive symptoms, as THC serves as a pro-psychotic agent that is prone to biological and social trends, and CBD has modulatory tendencies and neuroprotective features [15,16]. Premised on this multilevel model, the effects of cannabis on mental health cannot be generalized in the absence of an account of individual-level genetic endowment, neurochemical pathways, and social setting. This complexity highlights the need for a structured, evidence-based examination.

Thus, this systematic review seeks to assess recent peer-reviewed literature (2017-2025) to determine the involvement of cannabis in the onset and aggravation of symptoms of schizophrenia, as well as the therapeutic usage for schizophrenia. This review aims to integrate evidence from systematic reviews, meta-analyses, randomized controlled trials, and observational studies so as to assist public health decision-making, inform clinical decision-making, and contribute to a more granular understanding of the bi-dimensional psychiatric profile of cannabis [17,18].

## Review

The connection between cannabis and schizophrenia has been one of the most frequently studied topics in psychiatric research over the past 20 years. Owing to the increasing prevalence of cannabis use worldwide, especially in younger people, the neuropsychiatric effects of its psychoactive constituents have become increasingly recognized as the "Dual-aspect" of literature [19]. While THC has been vastly associated with an elevation in the risk for schizophrenia and exacerbation of its symptoms, CBD has been studied as an antipsychotic and has been proposed to be neuroprotective [20]. This review summarizes the current evidence in three thematic blocks: THC as a risk factor, THC on the course of the disease, and the therapeutic potential of CBD. 

Role of THC as a risk factor for schizophrenia

Substantial evidence supports the hypothesis that the use of the principal psychoactive constituent of cannabis, THC, increases the risk for and worsens the course of schizophrenia, particularly if used in adolescence. Prospective longitudinal cohort studies indicate that greater use, especially of high-THC varieties, is associated with an increased risk of psychotic outcomes [21,22]. These estimates were corroborated by a meta-analysis that indicated cannabis use is frequently associated with the incidence of psychosis in a dose-dependent manner [23]. From a neuroscience perspective, THC binds to the ECS, especially through the activation of CB1 receptors, which are involved in the dysregulation of dopaminergic and glutamatergic neurotransmitter systems, both of which are also important for the pathophysiology of schizophrenia. Genetic complexity is added to this relationship. COMT and AKT1 genes are significant modifiers of cannabis-induced psychosis risk. Individuals with these genetic types might be more susceptible to the neurochemical effects of THC, which translates into susceptibility to schizophrenia [24].

THC’s role in symptom progression and cognitive decline

In addition to the risk of precipitating schizophrenia, THC exacerbates the disease in patients diagnosed with schizophrenia. Several observational and clinical studies have also found that cannabis use, especially of high-potency THC, is linked to the development of increased positive symptoms (e.g., hallucinations and delusions), cognitive impairments, including working memory, attention, executive function, and processing speed, and treatment resistance [25]. Patients with schizophrenia who were regular users of cannabis required hospitalization more frequently and had worse long-term outcomes [26]. Mechanistically, THC acts on the cortical and subcortical regions to induce the activation of the CB1 receptor, resulting in perturbations in executive functioning, cognitive function, and emotional regulation. These effects could compromise adherence to antipsychotics and increase the risk of relapse. Interestingly, there are also data suggesting that THC can decrease the age of onset of psychosis, which is consistent with the hastened illness trajectory.

CBD and its newfound therapeutic potential

Unlike THC, CBD has been the focus of research in psychotic disorders, such as schizophrenia. Like THC, CBD does not interact directly with the CB1 receptors and has distinct pharmacological properties [27]. Preclinical and clinical studies indicate that the non-psychotropic CBD may serve as an allosteric antagonist of CB1, thereby dampening the psychoactive effects of THC, as well as modulating serotonergic (5-HT1A) and dopaminergic signaling [28]. CBD might reduce psychotic symptoms in patients with schizophrenia as well as have anxiolytic, anti-inflammatory, and neuroprotective properties. A clinical high-risk (CHR) for psychosis study in individuals at ultra-high risk (n=81) found large decreases in symptoms with short-term CBD treatment. Despite these promising results, the evidence base for CBD is limited by small sample sizes, heterogeneous dosages, and short follow-up intervals. Adding to this complexity is the prospect that CBD’s effectiveness may be linked to the subgroup of schizophrenia patients, illness stage, or genetic makeup of the patient, each of which has yet to be addressed systematically in the existing literature [29].

Integrative models and theoretical backgrounds

The literature has become more focused on the need for a more integrated understanding of the impact of cannabis on schizophrenia. Theoretical frameworks, which include biological, genetic, and social etiologies, provide more comprehensive predictions of the observed responses. For example, social factors such as childhood trauma, peer influences, and socioeconomic disadvantage may interact with biological vulnerability to mold the risk for cannabis-related harm [30,31]. This holistic framework transcends linear causality and emphasizes cannabis as a context-dependent agent, harmful in some instances (through THC) and possibly healing in others (through CBD) [32]. 

The written text alone cannot accurately establish the intended reading direction of the book. The current body of literature provides compelling evidence for THC as a risk factor and prodromal symptom promoter in schizophrenia. CBD, on the other hand, holds some early promise as a therapeutic agent but lacks strong clinical support to make definitive statements. Longitudinal investigations, genotype-stratified trials, and CBD-focused RCTs have been proposed to disentangle the compound-specific effects of cannabis in psychiatric conditions [33]. With increasing societal acceptance and medicalization of cannabis, defining these associations is imperative for clinical and public health.

Methodology

Review Design and Framework

This systematic review followed the Preferred Reporting Items for Systematic Reviews and Meta-Analyses (PRISMA) 2020 [34] guidelines. The goal was to conduct a critical review of the current literature (2017-2025) on the relationship between cannabis use and schizophrenia, addressing THC as a risk factor and CBD as a potential therapeutic agent.

Search Strategy

The following databases were searched: PubMed, PubMed Central, Cochrane Library, and Google Scholar. The search targeted peer-reviewed English-only articles published between January 2017 and April 2025. Research investigating the relationships between cannabis, especially THC and CBD, and schizophrenia/psychotic illnesses in humans was evaluated. The search was conducted using both mesh and free-text terms. Key words were “Cannabis”, “Marijuana,” “Schizophrenia,” “Psychosis,” “THC,” and “CBD,” and used the Boolean operators "AND" and "OR" as recommended for the search strategy in the databases. After removing duplicates, titles and abstracts of different studies were screened for the selection of studies for full-text review (Table 1).

**Table 1:** Initial search results across databases (2017–2025) MeSH: Medical Subject Headings; PubMed: U.S. National Library of Medicine Database; Cochrane: Cochrane Library (systematic reviews database).

Keyword/MeSH term	PubMed Central	PubMed	Google Scholar	Cochrane
Cannabis	14,000	1,350	67,500	37
Marijuana	22,000	1,670	48,800	67
Marijuana and Schizophrenia	4,570	124	14,700	1
Cannabis and Psychosis	2,460	73	5,540	1

Study Selection Criteria

A systematic literature search across PubMed, PubMed Central, Cochrane Library, and Google Scholar initially yielded over 82887 records. After filtering for duplicates, limiting to English language, publication years (2017 to 2025), and relevance to schizophrenia and cannabis (THC/CBD), a much smaller pool of 112 eligible records remained for screening. These 112 studies then underwent full-text review, quality assessment, and thematic categorization according to PRISMA 2020 guidelines. To improve methodological transparency, studies were required to meet the following minimum quality thresholds, based on the study design: (1) Systematic reviews/meta-analyses: at least seven of the 11 criteria on AMSTAR [35]; (2) RCTs: classified as “Low Risk of Bias” in a minimum of four out of five Cochrane domains; (3) Observational studies: a score of≥6 out of nine stars on the Newcastle-Ottawa Scale (NOS) and Narrative reviews [36], and a score of ≥10 out of 14 on the SANRA [37] scale. Studies that did not meet these criteria were excluded. Furthermore, case reports, editorials, animal studies, and gray literature were not considered. 

Inclusion Criteria

The inclusion criteria included systematic reviews, meta analyses, RCTs, observational studies, and high-quality narrative reviews. These were assessed using the SANRA tool, which required a minimum score of 10 out of 14, and were published in peer-reviewed journals. The study focused on people diagnosed with schizophrenia or psychotic disorders. Cannabis exposure was evaluated based on THC and/or CBD use. Lastly, the selection was restricted to studies published in English over the past eight years, from 2017 to 2025.

Exclusion Criteria

The exclusion criteria were non-peer-reviewed studies, animal-based publications, those with irrelevant schizophrenia, and research focusing solely on cannabis use disorder rather than psychotic outcomes. The inclusion and exclusion criteria are shown in Table 2.

**Table 2:** Inclusion and exclusion criteria for study selection RCTs: Randomized controlled trials; CBD: Cannabidiol; THC: Tetrahydrocannabinol.

Criteria	Inclusion	Exclusion
Study design	Meta-analyses, systematic reviews, RCTs, observational studies	Case reports, editorials, non-peer-reviewed literature
Publication date	Studies published from 2017 to 2025	Studies published before 2017
Language	English	Non-English publications
Population	Human subjects with schizophrenia or psychotic disorders	Animal studies, in vitro studies
Cannabis exposure	THC/CBD-focused studies	Studies on general substance abuse without schizophrenia focus
Therapeutic effects or outcome measure	The primary outcomes assessed included schizophrenia onset, symptom severity, cognitive impairment, and therapeutic effects (particularly the potential benefits of cannabidiol [CBD] in symptom management and neuroprotection	Studies lacking specific outcomes on psychosis
Peer review status	Published in peer-reviewed journals	Gray literature, conference proceedings
Methodological quality	Clear methods, valid statistics, reliable outcome reporting	Poor quality, high risk of bias

Quality Assessment of Included Studies

Two reviewers independently reviewed each study to maintain concordance and reliability. Differences in the assessments were reconciled by a third reviewer. Quality scores were assigned using the following standard tools. Systematic reviews and meta-analyses were assessed using AMSTAR 2 (scoring 0-11), RCTs assessment of randomization and blinding was performed using Cochrane Risk of Bias Tool [38], NOS for observational studies (score: 0-9 stars), SANRA for narrative reviews (score range: 0-14) and studies that scored above the minimum thresholds, as indicated previously, have reported on data extraction and synthesis. This strategy facilitated objectivity and consistency in the included studies.

Data Extraction and Synthesis

Data from each study were extracted using a standardized extraction form. The recorded information included the following: author(s), year of publication, study design, journal, characteristics of the sample (age, sex, population, symptom profile, if applicable), type of cannabis exposure (THC or CBD), dose, route of administration, and outcome measure (schizophrenia incidence, symptom severity, cognitive effects, treatment response, and hospitalization). The results section was structured by themes, which were formulated into three analytic categories: THC as a trigger or cofactor of schizophrenia, symptomatic progression and cognitive impairment, both effects of THC and CBD as therapeutics, and mechanisms underlying CBD’s effects.

Ethical Considerations

The present review did not include any direct involvement of human subjects and was based solely on publicly available peer-reviewed data. Ethical approval was not required because no personal or sensitive information was accessed during the review.

Conclusion to Review Methodology

This review was a structured, evidence-based investigation of the complicated interaction between cannabis and schizophrenia. By employing specific eligibility criteria, several quality assessment tools, and a thorough thematic synthesis indicated that the findings are credible and relevant. The review protocol not only guarantees scientific rigor but also serves as a framework to consider how THC may promote psychiatric liability and how CBD might offer therapeutic promise important distinction for both future research and public health policy and clinical practice.

Study Selection Overview

A systematic literature search across PubMed, PubMed Central, Cochrane Library, and Google Scholar initially yielded over 82887 records. After filtering for duplicates, limiting to English language, publication years (2017 to 2025), and relevance to schizophrenia and cannabis (THC/CBD), a much smaller pool of 112 eligible records remained for screening. These 112 studies then underwent full-text review, quality assessment, and thematic categorization according to PRISMA 2020 guidelines.

**Figure 2:**
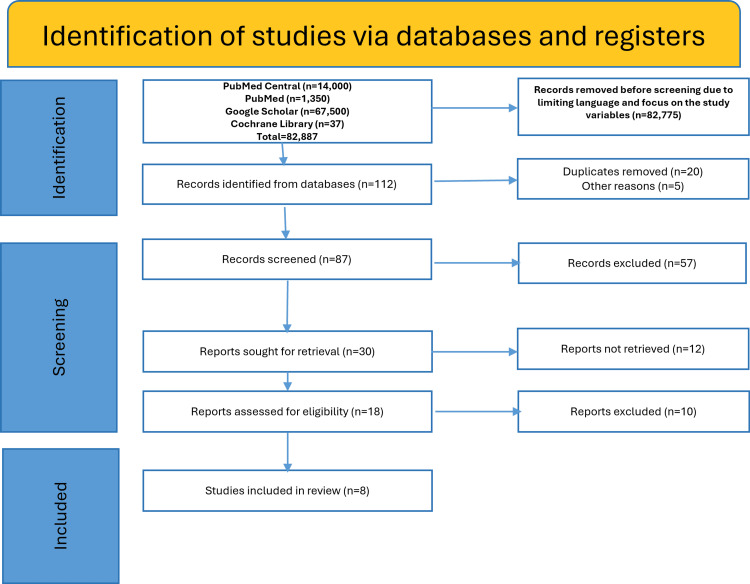
PRISMA flowchart PRISMA: Preferred Reporting Items for Systematic Reviews and Meta-Analyses.

Eight studies were eligible after removing duplicates and were screened for relevance according to the PRISMA 2020 criteria, as shown in Figure 2.

The current multidimensional synthesis of the relationship between cannabis and schizophrenia is based on these studies, which were standardized in terms of quality screening. 

As shown in Figure 2, out of the 82887 records retrieved from the initial search of the databases, a much smaller pool of 112 eligible records remained for screening. After removing duplicates or incomplete entries, 87 records remained. Out of these, 57 records were excluded after applying the inclusion criteria (due to wrong population, outcomes or study type). Out of 30 records sought for retrieval, 12 reports were not retrieved, as the full texts of the articles were not accessible or not retrievable. Finally, out of 18 records remaining, 10 were excluded due to poor quality assessment or their irrelevance to the outcome of schizophrenia, leaving a final count of eight studies to be included in this review [1-7,23].

Results

THC as a Neurobiological Risk Factor for Schizophrenia

Several recent reviews have presented strong evidence in favor of THC as a causative/accomplice pathogenic factor in schizophrenia [39]. THC acts on CB1 receptors, predominantly in the mesolimbic pathway, causing dopaminergic rushes that lead to hallucinations and delusions. Moreover, exposure of adolescents to THC (particularly those already genetically vulnerable (e.g., COMT Val158Met, AKT1 polymorphisms) increased the risk of psychosis. 

An evaluation of the eight studies included in the review indicated that reviews and meta analyses (n=5) showed moderate bias risk on AMSTAR 2, had some strengths, and included comprehensive searches of databases and dual screening, while lacking prescription protocols, publication bias, and definitions for exposures (e.g. cannabis potency, frequency of use) assessments. Genetically informed observational evidence (n=1) was classified as low to moderate on the NOS and strong bias controlling “confounding genetic instruments” but pleiotropy bias residual could not be excluded. 

The narrative evidence on CBD efficacy (n=1) had a moderate quality score on SANRA, which was adequate. The objectives were clear and the synthesis was well structured, but the control of the initial criteria was too loose. None of the included studies were RCTs, limiting the strength of claims concerning CBD's therapeutic effects.

As outlined in Table 3, THC’s action entails myriad changes in neurochemical measurements, from dysregulation of glutamatergic signaling to impairment of cognitive networks.

**Table 3:** THC vs. CBD: Mechanisms and effects CB1: Cannabinoid receptor type 1; 5-HT1A: 5-Hydroxytryptamine receptor 1A; CBD: Cannabidiol; THC: Tetrahydrocannabinol

Cannabinoid	Mechanism of action	Impact on psychosis	Cognitive effects	Clinical trial support	References
THC	CB1 receptor agonist; increases dopamine in the mesolimbic pathway	Increases risk; exacerbates symptoms	Impairment in memory and attention	Extensive, mostly shows risk and harm	[4,9,10,17,22]
CBD	5-HT1A partial agonist; increases anandamide; anti-inflammatory mechanisms	Potentially reduces symptoms; neuroprotective	Minimal impairment; may improve cognition	Limited, but promising early-phase results	[11,16,19,20]

**Figure 3:**
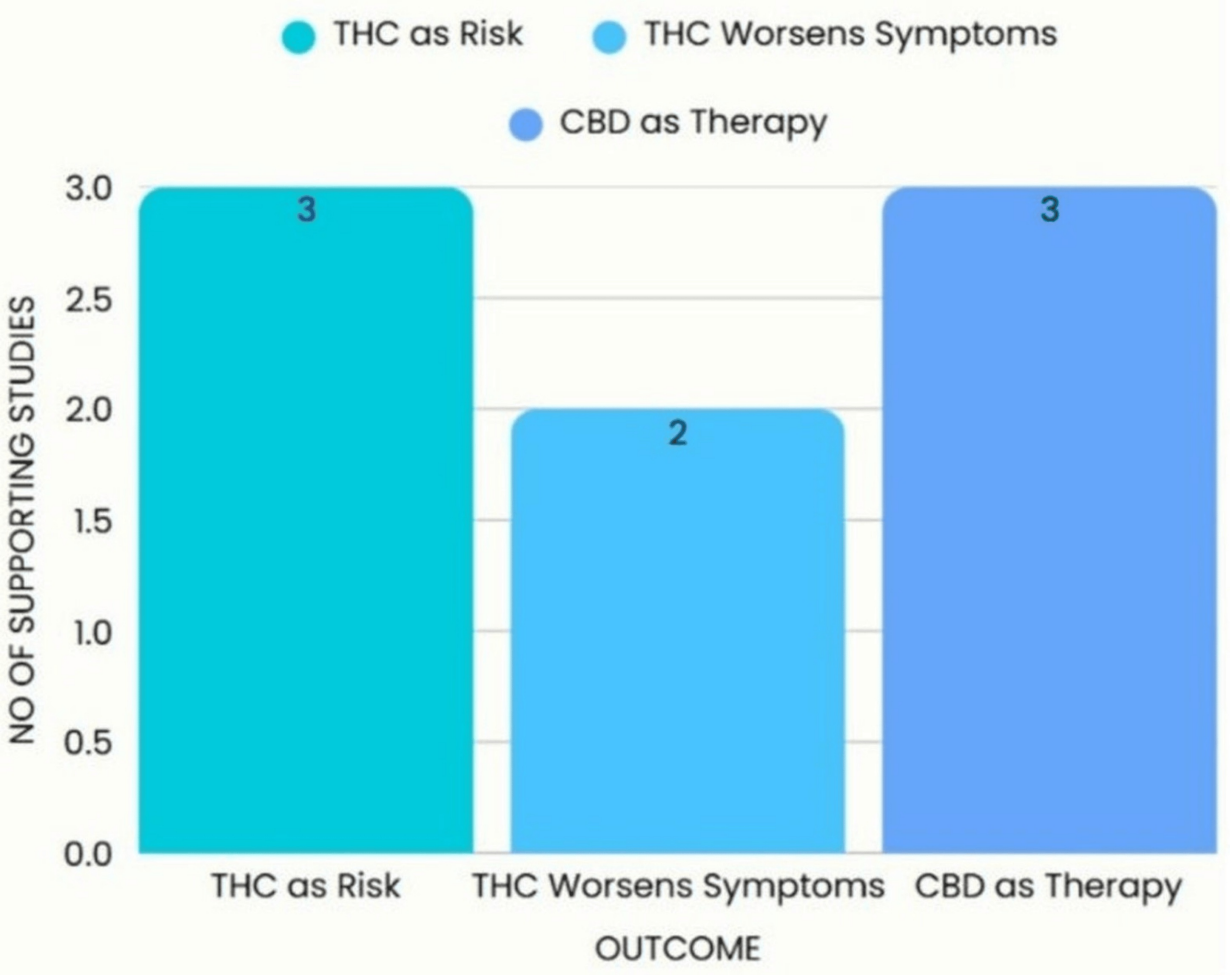
Number of studies supporting each outcome (2017–2025) CBD: Cannabidiol; THC: Tetrahydrocannabinol. Image created by the authors on Microsoft Powerpoint (Microsoft Corp., Redmond, WA, US) [3,7,8,27]

This data confirms that THC’s interaction with neural systems makes it particularly dangerous for at-risk populations. Figure 3 further emphasizes the concentration of research on THC’s causative role versus the emerging, yet underdeveloped, evidence base on CBD. 

Figure 3 shows that while CBD is a molecule with potential, evidence is lacking when compared to THC, which is more abundantly studied in the current literature.

THC and Aggravation of Symptoms in Diagnosed Subjects

High-potency THC use is linked to relapse, increased psychosis, and treatment resistance in individuals with schizophrenia. Regular use of THC was related to higher levels of positive symptoms (e.g., hallucinations, delusional beliefs) in the early onset of schizophrenia [40]. These clinical observation patterns are not coincidental and are consistent with neurochemical evidence, indicating that the dopamine system becomes dysregulated with repeated THC exposure [41].

Moreover, THC may interfere with working memory and prefrontal executive function, exacerbating the underlying cognitive deficits in schizophrenia. These results indicate that THC not only serves as an entry risk but also a perpetuating risk for this condition [42].

CBD: A Phenolic Compound with Promising Effects on Human Health

OAs opposed to THC, CBD has been studied as a neuroprotective and antipsychotic agent. Recent trials, such as the National Organization for the Reform of Marijuana Laws (NORML)-sponsored 2024 study, indicate that CBD can reduce positive symptoms in patients diagnosed with CHR for psychosis. CBD acts mechanistically by boosting anandamide signaling. (Note: Anandamide is often called the body’s own THC, functioning as a 5-HT1A partial agonist, antioxidant, and anti-inflammatory support). Although promising (Table 3), the mechanisms involved have not been investigated secondary to these being small, short-term ligament treatment clinical trials, and frequently non-comparative to an untreated control. For example, a 2023 BioMed Central (BMC) Protocol proposes a long-term RCT; however, no results have been reported. Thus, CBD could be an adjunct option (Undergraduate Thesis: Dobrić P, Interactions Between Cannabis and Schizophrenia, 2024).

Sociocultural Factors and the Self-Medication Paradox

Marijuana tends to be a form of self-medication. It is less about having a fun recreational experience, and more about treating anxiety, depression, trauma, and insomnia. This complicates the question of causality: Does cannabis cause psychosis, or are people who exhibit early symptoms more likely to use cannabis? Third, the social normalization of cannabis, including decriminalization and commercialization, might reduce the perception of its harm. High-THC products being available alongside the lack of public education increases the risk, particularly for adolescents who are already susceptible (Master’s Thesis: Legemaat S, Revisiting the Cannabis-Psychosis Association: Cannabis Use Does Not Cause Psychosis; 2024).

Comparative Outcome Synthesis

These findings are summarized by the type of outcome in the following table aggregating the volume and consistency of the evidence, as shown in Table 4.

**Table 4:** Summary of key outcomes (2017–2025) THC: Tetrahydrocannabinol; CBD: Cannabidiol.

Outcome	No. of studies supporting	Interpretation
THC as a causative/risk factor	3/5	Strong correlation with early use and genetic predisposition
THC exacerbating existing symptoms	2/5	Worsens psychotic symptoms and cognitive decline in diagnosed individuals
CBD as potential therapeutic compound	3/5	Shows preliminary promise; evidence still limited by trial design and scale

Conclusion of Analysis

The analysis from 2017 to 2025 depicts a distinct neurobiological and clinical dichotomy of THC and CBD. Whereas THC unequivocally appears as molecule whose exposure increases the risk of schizophrenia, biologically, socially, and genetically, CBD seems to be a fascinating yet under-validated candidate for treatment. 

Table 5 shows the PICO summary of included studies.

**Table 5:** PICO summary of the included studies THC: Δ9-tetrahydrocannabinol; CBD: Cannabidiol.

Study	Population (P)	Intervention / Exposure (I)	Comparator (C)	Outcome (O)
Patel et al., 2020 [1]	Patients with schizophrenia or psychotic disorders	Cannabis (THC/CBD) use	Non-users / general population	Cannabis may exacerbate schizophrenia symptoms; unclear therapeutic role
Kiburi et al., 2021 [2]	Adolescents	Cannabis use	Adolescents not using cannabis	Cannabis use linked with increased risk of psychosis; risk higher with genetic/environmental moderators
Masroor et al., 2021 [3]	Patients with schizophrenia + substance use disorder	Cannabis and other substance use	Non-substance users	Cannabis associated with worsened schizophrenia outcomes; dual diagnosis complicates treatment
Ahmed et al., 2021 [4]	Patients with schizophrenia	THC and CBD exposure	Schizophrenia patients not exposed	THC worsens psychosis; CBD shows preliminary promise for symptom reduction
Pourebrahim et al., 2025 [5]	General population (epidemiological focus)	Cannabis use	Non-users	Cannabis use significantly associated with schizophrenia incidence
Robinson et al., 2023 [6]	Cannabis users (varied frequency)	High-frequency cannabis use	Low-frequency or non-use	Psychosis risk rises with frequency of cannabis use (dose-response relationship)
Carvalho et al., 2022 [7]	Cannabis users with genetic polymorphisms	THC exposure	Cannabis users without high-risk polymorphisms	Genetic variants (e.g., COMT, AKT1) increase susceptibility to cannabis-induced psychosis
Groening et al., 2024 [23]	Mixed populations across psychosis continuum	Cannabis use (umbrella evidence map)	Non-use / lower exposure	Stronger evidence for THC-related psychosis risk; limited but emerging evidence for CBD benefits

The evidence is consistent with THC acting as a neurobiological risk factor for schizophrenia, with early and high-potency use hastening onset and compounding worse outcomes. CBD, on the other hand, is a candidate for a therapeutic adjunct, although its adjunctive role requires substantiation from carefully conducted clinical studies.

**Figure 4:**
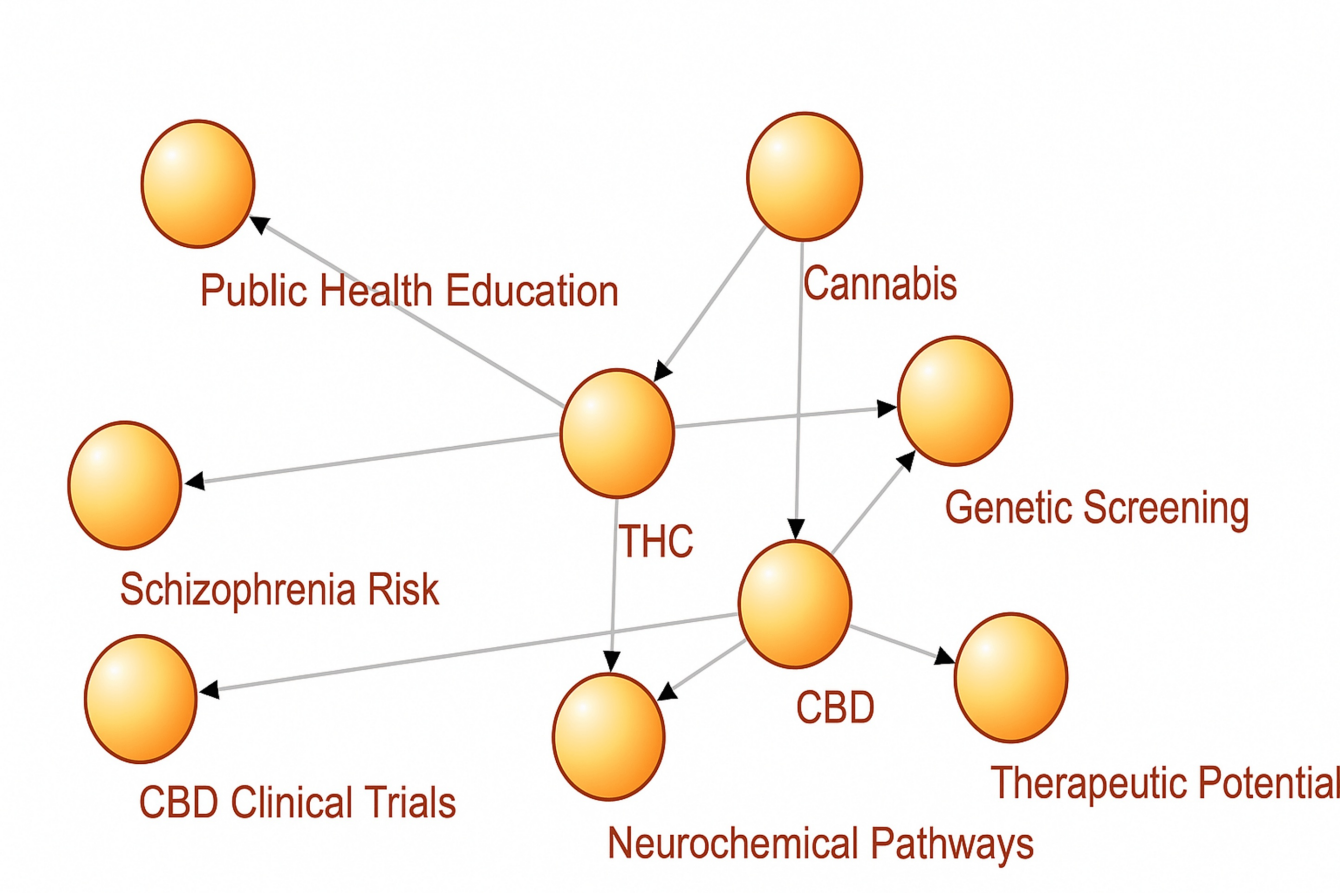
Biopsychosocial model linking cannabis to schizophrenia risk and therapeutic pathways CBD: Cannabidiol; THC: Tetrahydrocannabinol. Image created by the authors on Microsoft Powerpoint (Microsoft Corp., Redmond, WA, US) [27,28]

Neurochemical research on dopaminergic and glutamatergic systems and studies of known risk factors such as COMT and AKT1 gene variants and family history indicate the necessity of the population being educated about the topic of health (especially at a young age) and the need to conduct rigorous and evidence-based clinical trials on CBD [39]. Figure 4 shows the biopsychosocial model of how cannabis is related to the risk of schizophrenia along with its therapeutic pathways.

The model shown in Figure 4 depicts the dual-pathway administration of cannabis for schizophrenia. The model compartmentalizes cannabis into two main constituents: THC, exerting risk for raised schizophrenia rates in the context of neurochemical disturbance, genetic load (COMT, AKT1), and population risk exposure, and CBD, which is related to benefits in the schizophrenia spectrum, which should be corroborated in targeted clinical analyses. The model emphasizes the importance of a biopsychosocial approach in research examining the neurobiological, genetic, and environmental aspects of cannabis-related psychiatric outcomes. 

Discussion

This systematic study investigated recent peer-reviewed publications (2017-2025) to unravel the paradox of cannabis use and schizophrenia. Three main themes were identified: the role of THC in the onset and exacerbation of schizophrenia and its symptoms, the influence of cannabis use on the course of the disease and cognitive impairment, and the potential utility of CBD, a new player in the therapeutic arsenal. These findings add to a growing body of literature suggesting that cannabis should be conceptualized not simply as a single substance, but as a compound with specific data, and that individual cannabinoids differ in their psychiatric effects.

Role of Vulnerability and THC in Schizophrenia

This review supported the conclusion that there is strong evidence linking the use of high-potency THC with an increased risk of schizophrenia. Of crucial relevance to the problem under study is the fact that the risk is not the same in all populations. Rather, it is driven by biological vulnerabilities, such as pharmacological imbalance and genetic factors. Those exposed to THC in their teenage years, the period when marijuana use is most common and which corresponds with brain development, also seem to be especially at risk, and research has found these users to have both an earlier age of onset and a more severe presentation of psychosis. These findings are in accordance with the neurobiological model of schizophrenia, considering dysregulation (mainly in the mesolimbic system) of dopaminergic neurotransmission as a core neurobiological mechanism among patients with schizophrenia, THC exacerbates the positive symptoms of hallucinations and delusions, cognitive impairments, and increased rates of relapse and hospitalization. These results are corroborated by longitudinal epidemiological studies and clinical data, and imply that THC exposure not only induces but also accelerates disease progression in genetically- or environmentally-susceptible people [43,44].

CBD: A Potential But Inconclusive Medical Strategy

The review also noted increasing interest in CBD as an adjunct treatment for schizophrenia. In contrast to THC, CBD is non-psychoactive, and it has been suggested that it could be at least partly antipsychotic and neuroprotective. It may act, for example, by 5HT1A receptor modulation and inhibition of anandamide reuptake. Early phase clinical trials have demonstrated some attenuation of symptoms in symptomatic clinical high-risk individuals for psychosis or in those with early schizophrenia. Nevertheless, despite the promise of CBD, its therapeutic value is still premature, since most of the current trials are limited by small population sizes, short treatment periods, and lack of dose uniformity. Second, the extended use of CBD as a monotherapy or add-on antipsychotic treatment has not been demonstrated in large-scale placebo-controlled studies with follow-up for as long as one year (Master’s Thesis: Legemaat S, Revisiting the Cannabis-Psychosis Association: Cannabis Use Does Not Cause Psychosis; 2024).

Genetic Susceptibility: A More Refined Approach

Conversely, the AKT1 gene encodes a protein that is essential in the intracellular mechanisms of the dopamine cascade. Specific AKT1 polymorphisms (e.g., rs2494732) are associated with increased susceptibility to THC-induced psychosis. In contrast to COMT, novel evidence suggests that AKT1 may not only act as a modifier, but may also be a predictive biomarker for people who are biologically susceptible to psychosis following even lower levels of exposure to cannabis. It is unclear whether these genes play a causal role in the absence of marijuana. Recent research suggests that COMT (either genotype) or AKT1 (either genotype) alone is not sufficient to cause psychosis; therefore, these genes may have true etiological effects only when combined with frequent early exposure to THC or with younger age, increasing frequency, or high doses of exposure to THC, particularly for those with other vulnerabilities such as early trauma, social adversity, or pre-existing subclinical symptoms. This suggests a gene-environment interaction (Glee) model as opposed to direct causality [7,45,46].

Integrative Risk Framework: Cannabis, Genes, and the Social Environment

The risk of schizophrenia is influenced by a complex interplay of factors such as cannabis exposure (THC/CBD), genetic signature, neurobiological processes, and social milieu. This is because early use of cannabis, especially for teens growing up in socially deprived environments, can trigger dormant vulnerabilities in those who have genetic weaknesses. Trauma, marginalization, and poor access to mental healthcare are environmental triggers in this model, which amplify the expression of underlying biological vulnerability [47,48]. This review points to the need to refine the approach in future studies toward precision psychiatry, that is, to stratify participants based on genetic fingerprints (e.g., COMT, AKT1), measure exposure to THC and CBD, and thoroughly assess psychosocial background. This is the only way future research can disentangle real mediating variables and introduce preventive or interventional targets.

Implications for Policy and Practice

The independent discoveries of this review, THC as a risk factor and CBD as a possible therapeutic agent, hold important public health and clinical messages. Current legalization initiatives rarely consider harm related to high-THC cannabis, particularly for adolescents, young adults, and individuals with a family history of psychotic disorders. However, the clinical benefits of CBD merit further testing, although the therapeutic benefits should be exaggerated. Educational interventions should distinguish cannabis products from one another, and the fact that they are not necessarily all created equally in terms of potential harm or benefit. Practitioners should also regularly evaluate cannabis use history, such as the age at onset of use, product use, and frequency, especially in cases with early psychotic symptoms. For high-risk individuals who may carry nucleotide polymorphisms with COMT and AKT1 genetic variations, genetic testing may provide an important risk stratification and counseling tool if clinically validated in further studies.

The association between cannabis use and schizophrenia is one-dimensional. This review highlights the urgency of differentiating the psychiatric liability for THC from the potential therapeutic role and legacy of CBD, and argues for a more sophisticated appreciation of genetic vulnerability. COMT and AKT1 serve as nonspecific risk markers but as genetic modifiers and predictor genes, respectively, in a more complex gene-environment model of psychosis risk. By combining the biological, genetic, and environmental underpinnings of cannabis-related psychopathology into a common framework, our review shifts the discussion from mere associations to mechanistic understanding and ultimately toward increasingly personalized and evidence-based practice of cannabis-related psychiatry.

Limitations of the study

This review was constrained by the small number of eligible studies, and heterogeneity in study designs, populations, and cannabis formulations that limited comparability. Only peer-reviewed articles in the English language between 2017 and 2025 were included, introducing the potential risk of publication bias. The research on CBD is still preliminary; most trials were small, of brief duration, and lacked standardization of dosing. It was not possible to establish causality between cannabis use and schizophrenia because of self-medication effects and confounding variables. Interactions between genetic and environmental factors were inadequately examined, and limited longitudinal data limited the interpretation of long-term outcomes. Finally, the generalizability of findings is potentially restricted by socio-culturally determined and policy-specific differences between regions.

## Conclusions

This review analyzed the correlation between cannabis consumption and schizophrenia, including the contribution of THC to predisposing individuals to schizophrenia, particularly those at risk of being susceptible due to environmental and genetic vulnerabilities. Early or frequent exposure to the strong potency strain of THC is associated with an advanced onset of schizophrenia, cognitive delays, and increased cases of relapse and hospitalization.

In contrast, CBD, a non-intoxicating, cannabis-derived substance, demonstrates promise in treating the symptoms and stabilizing cognition, but there is limited evidence as to its long-term efficacy and safety. This review also looks into the effect of genetic factors, especially the variants of the gene coding the enzymes COMT and AKT1, which interact with THC exposure to modulate psychosis. This implies that the effects of cannabis use on schizophrenia vary with the substance used as well as with genetic and environmental factors. It also underscores the importance of further targeted research into this illness and the creation of patient-centric treatment strategies.
